# Family life at Okapa as a ‘missus bilong dokta bilong kuru’

**DOI:** 10.1098/rstb.2008.4019

**Published:** 2008-11-27

**Authors:** Coralie Mathews

**Affiliations:** 19 Earl StreetCarlton North, Victoria 3054, Australia

This 50th anniversary of the first diagnosis of kuru brings back so many memories and these reminiscences are for all the ‘kuru wives’, especially Wendy Alpers and Fay Hornabrook.

John and I went to live in the Fore area early in 1966. It was expected that we would live at the government administration centre of Okapa as John, we found on arrival, was often required to double as the medical officer for the Okapa Subdistrict. We did sometimes manage to escape the colonial pressure and spent periods living in the Glasse's house in Wanitabe and in the kiap's hut (house built for the use of patrol officers on patrol) at Purosa.

Travelling to and from Okapa with a young family was always exciting, and often totally worrying! From Goroka, you would take a single-engine light aircraft that bobbed between the mountains. Sometimes there would be no seat for you and you would be squashed on the floor (no seat belt and knees tucked under your chin) in the tail of the plane behind all the cargo, which was often made up of Carleton Gadjusek's equipment!

Road travel was by army-style four-wheel-drive vehicles that slowly edged their way along the narrow ledges of the steep valleys and bumped through river crossings not infrequently getting bogged. Monday was not a good day to travel because each village was required to work on their part of the road on that day (figure 1). Many of the roads were a pile of rocks with bridges reduced to a few planks. This meant lots of forced stops where you took the opportunity to buy kaukau (sweet potato) and small sweet pineapples from the women in the nearest village.

Travel between villages and hamlets was by walking along narrow paths between the kunai grass and over stiles built along the fences of diwai (wood) and bamboo, which kept the pigs out of the gardens. And much of the walking was up and up. I can remember going to Mugaiamuti early one Sunday morning to help with an autopsy and being pulled up the steep muddy track with a stick of bamboo held over the shoulder of a sturdy and helpful local kuru assistant.

Single-log bridges over gushing rivers were a fearful ordeal for me. I would give the baby and toddlers to the sure-footed local guys who would whisk the children away to the other side of the ravine in no time. I would then falteringly and fearfully slowly step across sideways like an injured crab clinging to a patient helper on each arm. I probably whimpered all the way as well!

The settlement at Okapa was a physically beautiful place that looked out over a long valley with the village of Pusarasa nearby and Mt Michael in the distance (figure 2) jutting his (this mountain was definitely male) head out of the clouds that filled the valley. It was my morning ritual to greet the tip of Mt Michael as I stumbled to the kitchen and the warmth of a wood fire.

In the early evenings, the women of Pusarasa would pass through, laden with kaukau, firewood and small children, on the way from their furthest gardens. They would call out and chatter, whereas the men carrying axes were much more serious. By this time, the axes were bought at the trade store and not the stone axes used in the very recent past.

A group of Australian wives started working with the local women. There were many Fore women who walked in from their villages to talk about child health and a tentative start was made, at their request, with a family planning clinic. The local women were very enthusiastic and vocal about these issues. We also had fun playing baseball and other games. Local crafts were displayed and there was much excitement when our women won first prize in the Goroka Show.

The mid-1960s was the last of the colonial era: a period of Pax Australiana. The Australian flag flew proudly over the kiap's corrugated iron offices (a kunai grass hut would never have done) and the sun never set upon it. The flag also rose at dawn as I discovered one misty cold morning when walking back from helping with an emergency at the hospital. There alone in his uniform, a policeman, from the Manus islands and posted to Okapa, played his bugle and raised the flag! No one but he and I heard it that morning and for most sunrises he was alone!

Kuru pervaded all parts of our lives. All the Fore people who worked at Okapa, who helped in our house or who played with our children, had close relatives who had died or would die of the disease.

There was gentle Yat, a young teenager, whose mother would soon die and then, some years later, Yat also. Hani's sister who lived at Paigatasa was dying from kuru. He took the long walk home with John and Mark (our son) to say goodbye (figure 3). Abote's wife with a very young baby was already dying when the baby was born. There were the trips to villages to dress pressure sores to try to ease the pain…but nothing could be done to treat the disease.

The orphanage at the Lutheran Mission at Awande was a stark reminder of the emotional deprivation caused by kuru. Babies and toddlers, too small to get adequate protein from kaukau, were taken to the orphanage with an older child as their carer. The carers were approximately 8–10 years of age and they provided adequate physical care and fed the younger children with milk and weaning foods provided by the missionaries. But the carers wanted to play with each other and were too young to provide emotional care for infants and toddlers. So their charges would spend hours sitting passive and alone while the sound of excited play was heard in the distance.

This was the legacy of kuru that I saw in the 1960s: the loss of loved ones and the sadness of so many babies and young children.

## Figures and Tables

**Figure 1:**
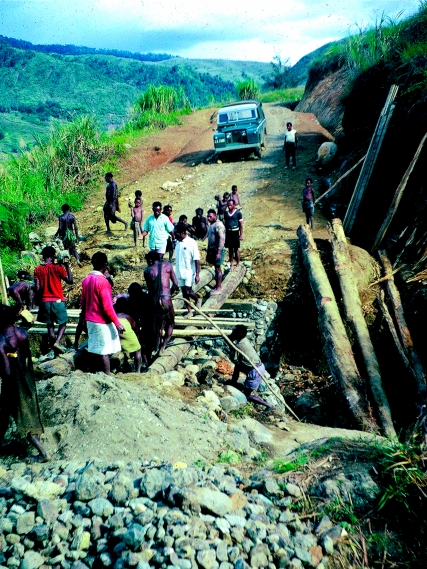
Getting to Okapa by road on a dry day: bridge under repair by a working party from a village nearby.

**Figure 2:**
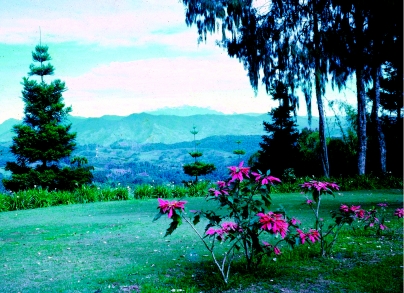
Mt Michael from the front garden of our house in Okapa—an ever-changing view.

**Figure 3:**
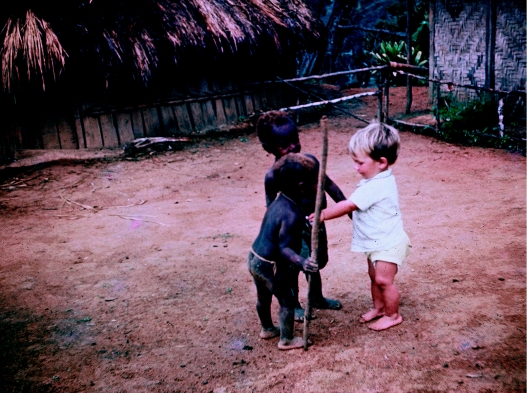
Mark playing with friends at Paigatasa.

